# Embroidered Textile Antennas: Influence of Moisture in Communication and Sensor Applications

**DOI:** 10.3390/s21123988

**Published:** 2021-06-09

**Authors:** Davor Bonefačić, Juraj Bartolić

**Affiliations:** Faculty of Electrical Engineering and Computing, University of Zagreb, Unska 3, HR-10000 Zagreb, Croatia; juraj.bartolic@fer.hr

**Keywords:** textile antenna, embroidered antenna, patch antenna, PIFA, wideband planar monopole, moisture, waterproofing, moisture sensor

## Abstract

Moisture causes detuning and increased losses in textile antennas, and it affects resonant and wideband textile antennas differently. In this work, we studied the effect of moisture on a resonant textile planar inverted-F antenna (PIFA) and a wideband textile monopole antenna. Both antennas were manufactured by embroidering conductive yarn in denim textile. The input reflection coefficient, antenna gain, and gain patterns were measured on both antennas for different moisture contents. The results show that wideband antennas are less affected by moisture in comparison with resonant antennas. For communications applications, large moisture content in the textile antenna should be avoided; therefore a flexible, textile-based waterproofing antenna cover was proposed, manufactured, and tested. On the other hand, the effect of antenna detuning by moisture can be used for moisture-sensing application. This concept was demonstrated on the resonant textile PIFA in transmission and reflection setups, showing that the reflection setup gives better results.

## 1. Introduction

Modern personal communications, wireless sensors, and other wireless devices need a large number of antennas to be connected to wireless networks or to the Internet-of-Things. Antennas are presently, and in the future will inevitably be, integrated in many different devices, as well as on the human body and on clothes [1]. Such antennas have to be lightweight and flexible. Common materials such as metals and dielectrics used for manufacturing of conventional antennas have to be replaced with conductive and non-conductive textiles and yarn to allow seamless integration with garments. A full-textile antenna is an antenna manufactured solely out of conductive and non-conductive textiles and yarn.

Conductive and non-conductive textiles are porous, unfortunately; hence, water and water vapor easily penetrate into the material, which results in increased losses and changes in the material dielectric properties. This in turn results in a change of the input impedance, gain, and radiation patterns of the textile antenna.

## 2. Description of Textile Antennas

The objective of this work was to investigate the influence of moisture on embroidered textile antennas. In contrast to Reference [2], where various substrate materials were considered, this paper focuses on different types of embroidered textile antennas. A shorted quarter-wavelength long patch, namely a planar inverted-F antenna (PIFA), was selected as a representative of resonant antennas, and a wideband textile planar monopole was used as representative of wideband antennas. Both of these antennas were full-textile antennas; namely, the substrates were made out of textile, while the conductive parts were made by embroidering of conductive yarn.

The design of the textile PIFA was presented in References [3,4] in more detail. For completeness, a short outline is given here. The PIFA was designed as a shorted quarter-wavelength-long microstrip patch by using CST Microwave Studio [5]. The design goal was the operation in ISM 2.4 band. Optimization in the CAD tool gave the initial antenna dimensions. However, in the simulated model, both the patch and the ground plane were represented as solid conductive plates, as modeling of the exact embroidered threads would prohibitively increase the complexity of the model and thus the computation time. Therefore, the final dimensions obtained by simulation had to be adjusted and experimentally tuned to take in account the effect of the lockstitch used for embroidering [3,4].

**Figure 1:**
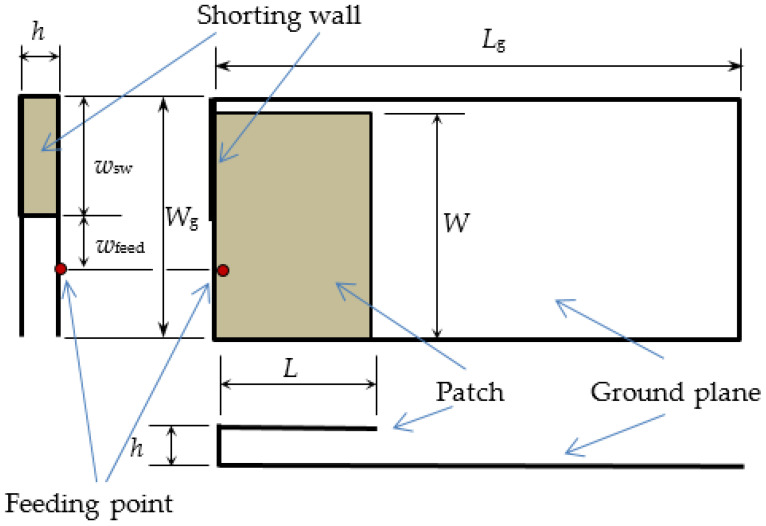
Side, top, and front view of the PIFA.

**Figure 2:**
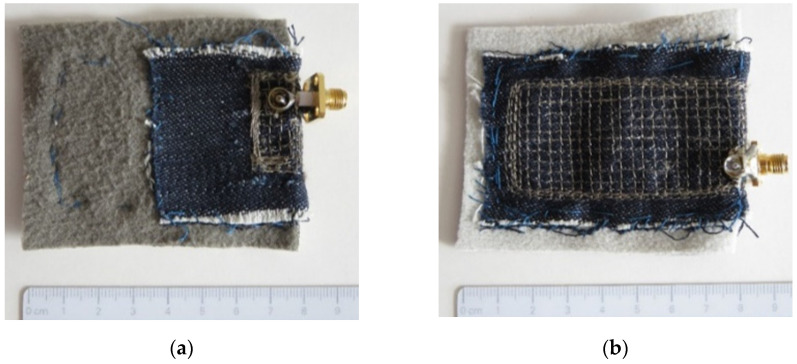
Textile PIFA prototype: (**a**) patch and (**b**) ground plane.

The drawing of the PIFA is shown in Figure 1, and the dimensions of the manufactured textile prototype (shown in Figure 2) are patch *W* × *L* = 30 mm × 16 mm and ground plane *W*_g_ × *L*_g_ = 32 mm × 70 mm. The shorting wall width is *w*_sw_ = 16 mm, and the feed position is *w*_feed_ = 7 mm. The antenna height is *h* = 4 mm. The conductive parts (patch, ground plane, and shorting wall) of the PIFA were realized by embroidering rectangular mesh of conductive threads [6] in denim textile rather than by dense embroidering. The general size of the mesh was 3 mm × 3 mm. Denser mesh was used at locations of higher current density (e.g., edges) predicted by the CAD tool (Figure 2). The substrate is realized by one layer of fleece with *ε*_r_ = 1.17, resulting in total antenna thickness equal to *h* = 4 mm.

**Figure 3:**
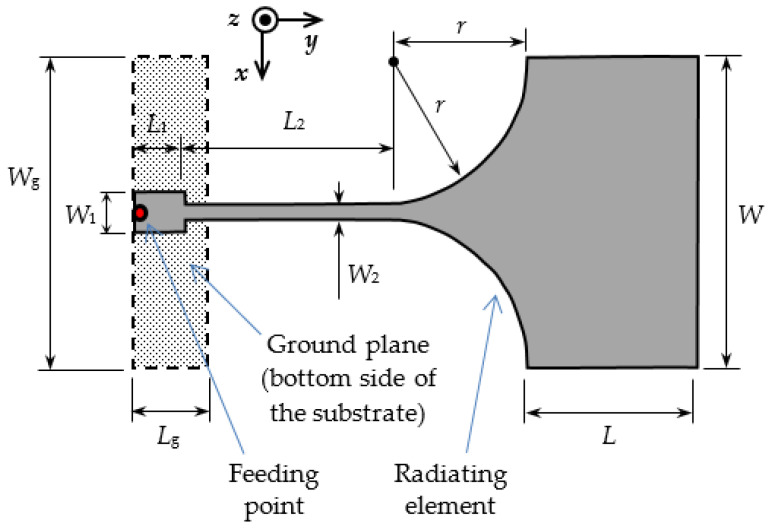
Textile planar wideband monopole; the radiating element is on the top and the ground plane on the bottom side of the textile substrate. The appropriate coordinate system for the radiation patterns is shown on the top middle of the figure.

**Figure 4:**
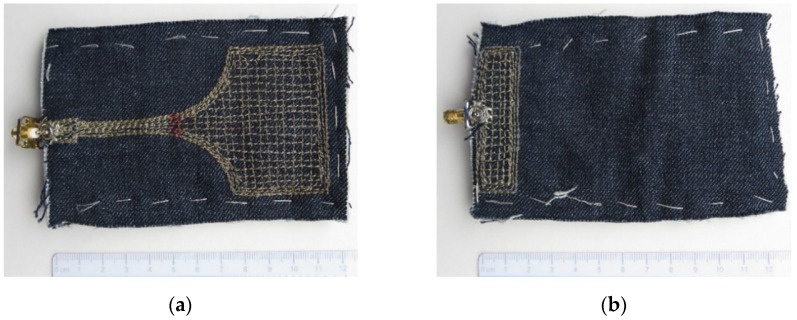
Wideband textile planar monopole: (**a**) radiating element and (**b**) ground plane.

The wideband monopole design was inspired by References [7,8,9]. The drawing of the resulting monopole is shown in Figure 3. The design intention was to obtain an antenna operating both in 2.4 and 5.8 GHz ISM bands. Extensive parametric studies for the proposed design are documented in Reference [10]. Optimization was again performed by using CST Microwave Studio [5]. The wideband characteristic of the monopole is realized by two resonant frequencies of the structure in Figure 3 with overlapping bands. The resonant frequencies are tuned by the dimensions *W* and *L* of the radiating element, while the input impedance matching in the desired bandwidth is obtained by adjusting the dimensions of the feeding lines *W*_1_, *L*_1_, *W*_2_ and *L*_2_, as well as the length of the ground plane, *L*_g_. As each tuning is not independent from the other, an iterative tuning procedure was used. The final dimensions of the realized textile monopole are radiating element *W* × *L* = 60 mm × 38 mm and ground plane *W*_g_ × *L*_g_ = 60 mm × 16 mm. The dimensions of the feeding lines and taper are *W*_1_ = 10 mm, *L*_1_ = 14 mm, *W*_2_ = 6 mm, *L*_2_ = 36 mm and *r* = 25 mm. The substrate height is *h* = 4 mm. The photos of the radiating element side and ground plane side of the manufactured textile monopole are shown in Figure 4. The monopole and the ground plane were realized by embroidering conductive yarn [11] in a 3 mm × 3 mm mesh. Embroidering was done on denim textile, while the substrate of thickness *h* was realized by using one sheet of fleece. Again, the mesh density was increased at the edges of the radiating element and on the feeding line, i.e., at the locations where larger current density was predicted by simulation.

For testing purposes, both antennas, the PIFA and the monopole, are excited by an SMA connector placed in the plane of the antenna. As both conductive yarns [6,11] used for antenna manufacturing cannot withstand temperatures used for soldering, the transition between the embroidered conductive patterns and the classic SMA connector was realized by snap-on buttons regularly used in clothes manufacturing. One part of the snap-on button was sawn on the embroidered pattern by conductive thread, while the other was soldered to the connector [3,4,12].

## 3. Measurement Results

The moisture content in an object expressed in percent *M*(%) is given as follows:

(1) $$M(\%)=\frac{\;m_{\mathrm{wet}}-m_{\mathrm{dry}\;}}{m_{\mathrm{dry}}}\cdot 100\%,$$

where *m*_dry_ is the weight of the considered object when it is dry, while *m*_wet_ is the weight of the same object when it has absorbed some moisture, i.e., when it is wet. In (1) the weight of the dry object (*m*_dry_) is used as reference, hence the obtained moisture content *M*(%) is the moisture content on dry basis. This definition of moisture content is used throughout the paper.

The discrete values of moisture content in the textile antennas during measurement were obtained by spraying the antennas under test with fine water mist on both sides (top and bottom) to ensure uniform water absorption in the textile. When required, the antennas were dried by hot air. The moisture content in the antenna was determined by measuring its weight by a precision scale with a resolution of 0.01 g. The weight of the SMA connector was subtracted from all the measured weights prior to moisture content computation. The input reflection coefficient, gain and radiation patterns for both textile antennas under test were measured for every 10% increase in moisture content from 0% to 100% and additionally for 5%, 25% and 75%. Preliminary measurement results were reported in References [13,14]. In the next sections, we provide more details and a discussion of the results.

### 3.1. Measurement Results for the Textile PIFA

**Figure 5:**
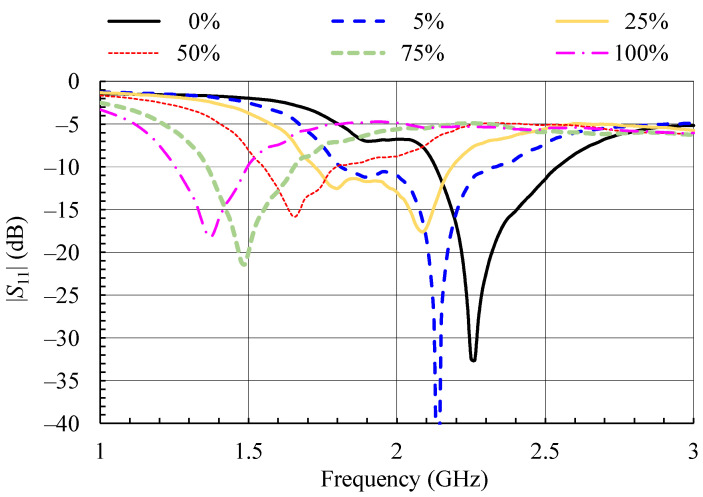
Measured magnitude of the input reflection coefficient of the textile PIFA for different moisture contents [13,16].

The input reflection coefficient of the PIFA was measured for different moisture contents. The measurement results, given in Figure 5, show that the increase of the moisture content in the textile material shifts the resonant frequency of the PIFA towards lower values.

The reference in Figure 5 is the plot for 0% moisture. It shows that the PIFA is well matched in ISM 2.4 frequency band. A 5% increase in moisture content shifts the resonant frequency of the PIFA from 2.2625 to 2.1375 GHz, namely, by 5.5%. At 25% moisture content, another, lower resonance is formed (Figure 5). By further increasing the moisture content the lower resonance becomes dominant and further decreases in frequency with moisture content increase. At moisture content of 100%, the resonant frequency of the PIFA is shifted for more than 39%, i.e., to 1.3750 GHz.

The conclusion of the experiment is that even small moisture content (about 5%) detunes the PIFA sufficiently to reduce the match (|*S*_11_| > −10 dB) in the original frequency band (ISM 2.4). This is in agreement with results in References [2,4,15].

**Figure 6:**
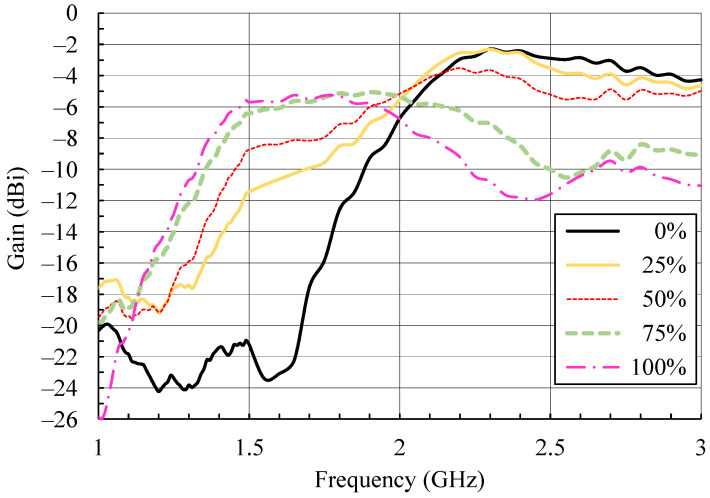
Measured gain of the textile PIFA for different moisture contents [13,16].

To investigate the influence of the losses introduced by moisture, the gain of the PIFA at 0° (direction perpendicular to the plane of the antenna) was measured, and the results are shown in Figure 6. A large frequency span from 1 to 3 GHz was chosen to include all the frequencies to which the PIFA is detuned due to moisture.

The reference plot for 0% moisture in Figure 6 shows that the dry PIFA at resonance has a gain of −2.7 dBi. This is about 3 dB lower in comparison to the gain of an equivalent metallic PIFA [3]. The lower gain of the textile antenna is caused by the conductivity of the yarn used for embroidering that is about two to three orders of magnitude lower in comparison to the conductivity of solid copper. The PIFA gain remains largely unchanged up to moisture content around 50%, beyond which it appears to monotonically reduce and reaches −10.5 dBi at 100% moisture content. The conclusion based on these measurements is that the main effect of increased moisture is that it detunes the textile resonant antenna, while antenna gain reduction becomes significant only with larger moisture content.

Furthermore, it should be noted that, at frequencies where the dry PIFA is not radiating (e.g., around 1.5 GHz) and the gain is negligible (<−20 dBi), increased moisture content allows for considerable increase in radiation. Between 75% and 100% moisture content in the frequency band around 1.5 GHz, the antenna gain increases to about −6 dBi, which is only 3 dB lower relative to the gain of the dry antenna in its operating band. Furthermore, for 75% moisture content, the PIFA is also impedance matched at its input at 1.5 GHz (Figure 5). This effect is, however, not appropriate for communication applications, but it reveals the potential to use resonant textile antennas exposed to moisture as moisture sensors, which is discussed in Section 5.

**Figure 7:**
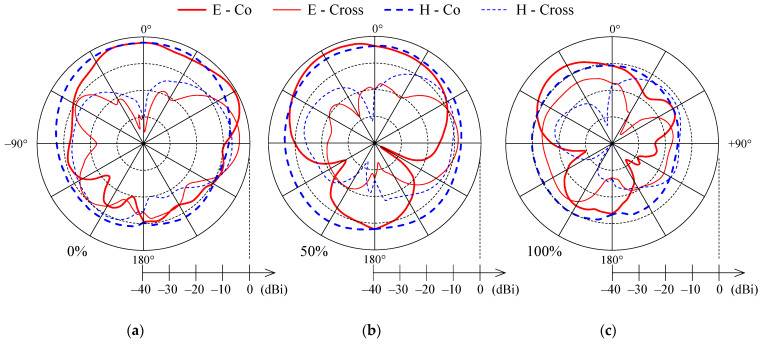
Measured co-polarization (thick line) and cross-polarization (thin line) gain patterns in E-plane (solid) and H-plane (dashed) for the textile PIFA with (**a**) 0% moisture content, (**b**) 50% moisture content, and (**c**) 100% moisture content.

Gain patterns in all cases were measured at the resonant frequency of the dry PIFA. The gain patterns for the dry PIFA and for moisture contents of 50% and 100% are in Figure 7. From Figure 7, one can observe that the shape of the gain co-polarization patterns in both planes does not change significantly with increased moisture content. At 50% moisture content, the back lobe in E-plane becomes more noticeable, and some increase in the cross-polarization levels with increasing moisture content can be observed. Of course, all the patterns shrink towards the center (Figure 7) as gain decreases with increased moisture content.

### 3.2. Measurement Results for the Textile Monopole

**Figure 8:**
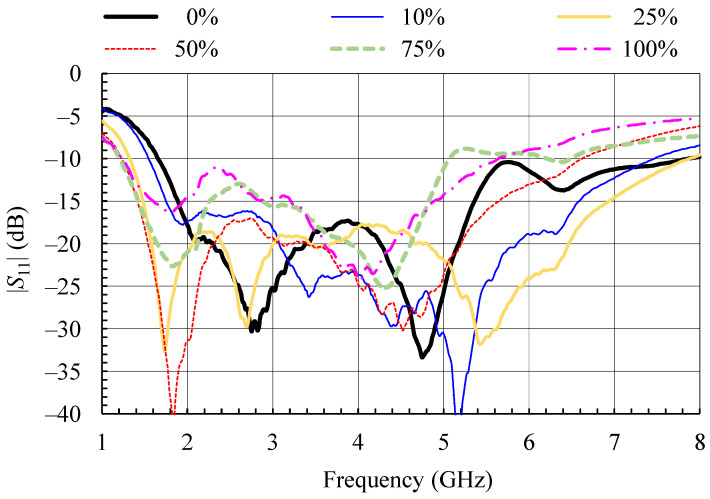
Measured magnitude of the input reflection coefficient of the wideband textile monopole for different moisture contents [14].

The results for the measured input reflection coefficient of the wideband textile monopole for different moisture contents are shown in Figure 8. The reference is the plot for 0% moisture. For the dry case, the textile monopole has good impedance matching (|*S*_11_| < −10 dB) in the band from 1.665 to 7.860 GHz, so the design goal of operation in both ISM 2.4 and 5.8 bands was met.

Nearly the same operating bandwidth as for the dry case is maintained for moisture content up to 25%. A further increase in moisture content results in increased reflection at higher frequencies and, with it, in bandwidth reduction, while, at lower frequencies, the impedance matching bandwidth is extended. Therefore, the impedance matching bandwidth is again shifted towards lower frequencies with increased moisture content. Regarding the input impedance matching and its bandwidth, the considered wideband monopole can be used for all considered moisture contents with minor degradation in performance at the upper bandwidth end.

**Figure 9:**
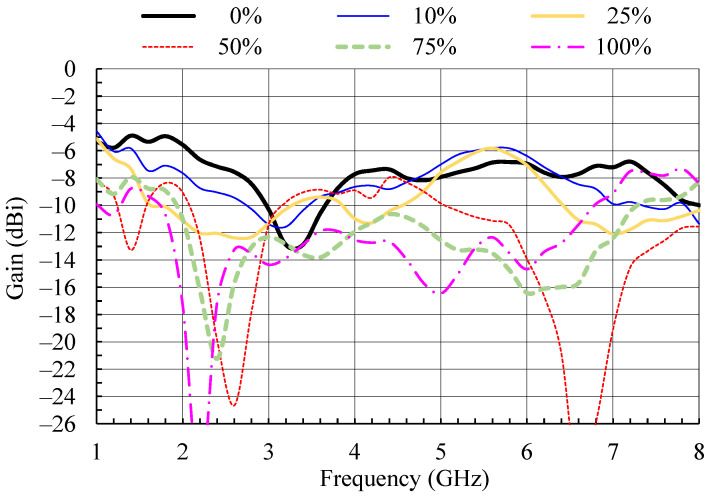
Measured gain of the wideband textile monopole for different moisture contents [14].

**Figure 10:**
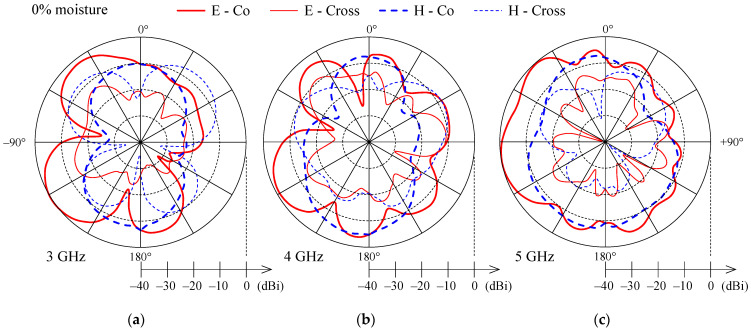
Measured co-polarization (thick line) and cross-polarization (thin line) gain patterns in E-plane (solid) and H-plane (dashed) for the wideband textile monopole with 0% moisture content at (**a**) 3 GHz, (**b**) 4 GHz, and (**c**) 5 GHz.

**Figure 11:**
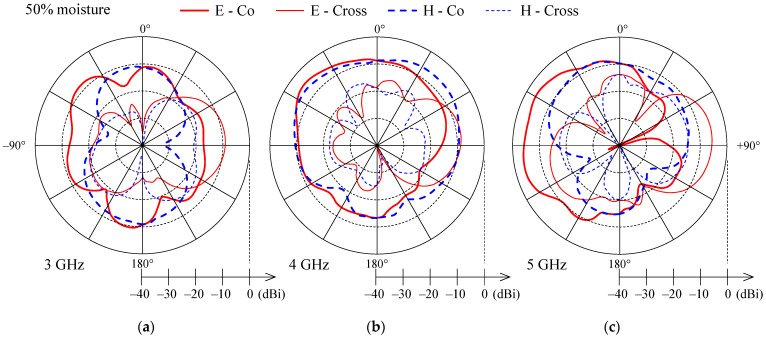
Measured co-polarization (thick line) and cross-polarization (thin line) gain patterns in E-plane (solid) and H-plane (dashed) for the wideband textile monopole with 50% moisture content at (**a**) 3 GHz, (**b**) 4 GHz, and (**c**) 5 GHz.

**Figure 12:**
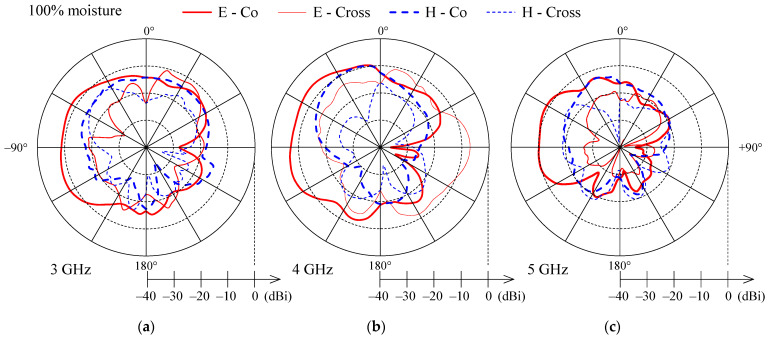
Measured co-polarization (thick line) and cross-polarization (thin line) gain patterns in E-plane (solid) and H-plane (dashed) for the wideband textile monopole with 100% moisture content at (**a**) 3 GHz, (**b**) 4 GHz, and (**c**) 5 GHz.

To verify that good impedance matching at the monopole input is not just a result of increased losses caused by moisture, the antenna gain and gain patterns as functions of frequency and moisture content were measured (Figure 9, Figure 10, Figure 11 and Figure 12). The gain in Figure 9 was measured in the *z*-axis direction (Figure 3). As the conductivity of the yarn [11] used for embroidering the textile monopole is more than two orders of magnitude lower in comparison to the conductivity of a good conductor, such as copper, the gain of the dry textile monopole is below 0 dB in the whole bandwidth (Figure 9). Moisture contents increase up to 25% results in antenna gain reduction of up to 6 dB in comparison to a dry antenna. Nevertheless, the monopole antenna performance is acceptable for use in a wireless communication system, but with reduced range. At a few frequencies, at moisture contents of 50% and above, notches of more than 10 dB in gain values were observed (Figure 9). A wideband wireless system would not be severely disturbed by this effect, but the operation of narrow-band systems in that bands would be disrupted, or the range would be severely reduced.

Figure 10, Figure 11 and Figure 12 present the measured co-polarization and cross-polarization gain patterns of the wideband textile monopole for the dry antenna (0% moisture) and 50% and 100% moisture content. The gain patterns are measured at three different frequencies in the monopole operating band, i.e., at 3, 4, and 5 GHz. The E-plane corresponds to the *y*–*z* and the H-plane to the *x*–*z* planes according to Figure 3.

For the dry case (0% moisture), the E-plane co-polarization patterns (Figure 10) show several lobes and nulls which is a result of radiation from the monopole, the feeding lines, the ground plane, and the connector. With increasing frequency (Figure 10) the null depth is reduced and the E-plane co-polarization patterns are smoother. With increased moisture content (Figure 11 and Figure 12) the lobes and nulls in the E-plane co-polarization patterns are also reduced. The reduction of null depth in E-plane co-polarization radiation patterns at higher moisture content is explained by increased losses due to moisture which reduce unwanted radiation from the feeding lines and ground plane.

H-plane co-polarization gain patterns for 0%, 50% and 100% moisture contents (Figure 10, Figure 11 and Figure 12) show nearly omnidirectional radiation. Cross-polarization levels (Figure 10, Figure 11 and Figure 12) are high and, at some off-broadside angles, exceed the co-polarization ones. However, for antennas which will be integrated in clothes and used on the user’s body, this should not be a drawback, as body movement, antenna bending, and crumpling, as well as complex propagation environment, will anyhow affect the polarization of the radiated and received wave.

## 4. Waterproofing

In an on-body or off-body communication system wearable textile antennas integrated in clothing will be exposed to moisture from outside (e.g., rain), as well as from inside (e.g., perspiration). As moisture content (especially above 50%) degrades the properties of both considered antennas, we investigated the possibility of waterproofing the antennas. Direct application of a waterproofing coat on the textile antenna can chemically interact with the conductive yarn or penetrate in the textile substrate and change its properties. Therefore, we decided that applying a waterproofing cover was a better approach.

**Figure 13:**
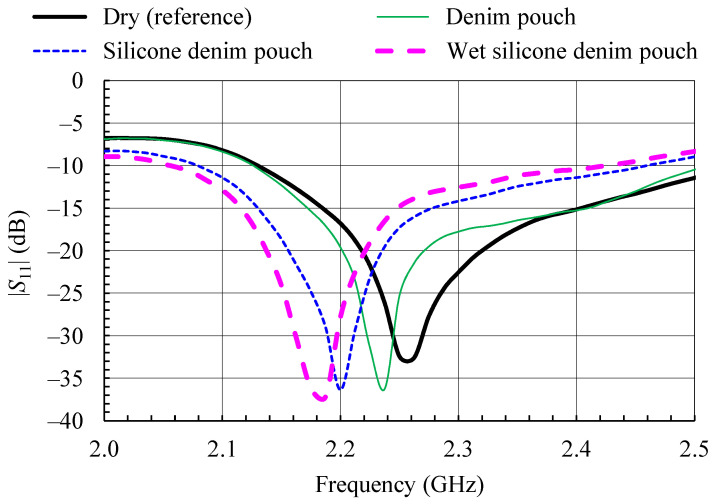
Measured magnitude of the input reflection coefficient of the textile PIFA with protective silicone-sealant-coated denim pouch.

**Figure 14:**
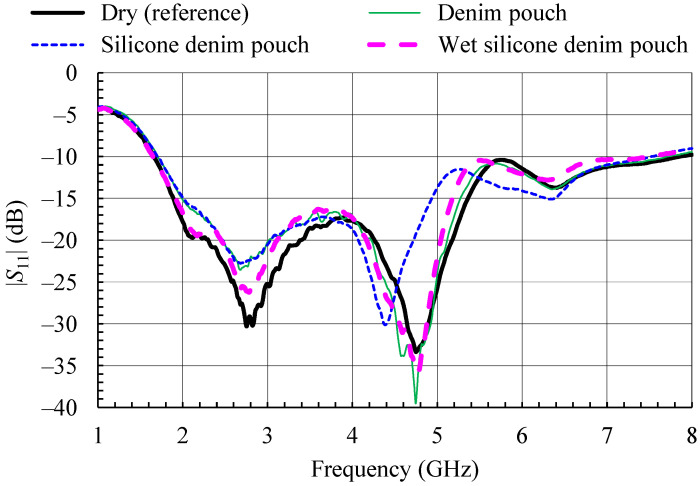
Measured magnitude of the input reflection coefficient of the wideband textile monopole with protective silicone-sealant-coated denim pouch.

The waterproofing cover is realized in form of two pouches, one for each of the considered antennas, made of the same denim material as the one used for the antennas. The pouches were made waterproof by applying a thin (<1 mm) layer of silicone sealant to the outer surface. Each pouch completely encloses the antenna, and only the feeding RF cable passes through. The measured reflection coefficient magnitudes for both the antennas under test in silicone-sealant-coated denim pouches (dry and wet) are shown in Figure 13 and Figure 14. For comparison, Figure 13 and Figure 14 show also the reflection coefficient magnitude of the dry antennas (PIFA and monopole, respectively) without the pouch, as well as the reflection coefficient at the input of the antennas in the denim pouch before the application of the silicone sealant layer. Additional dielectric layers over the PIFA resulted in slight detuning (Figure 13), while the influence of the waterproofing layers on the wideband monopole is almost negligible (Figure 14).

**Figure 15:**
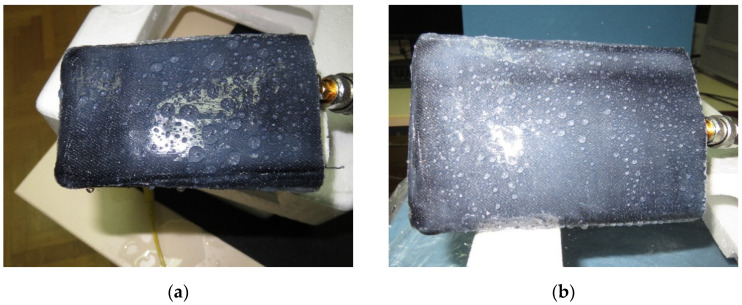
Water droplets formed on the protective silicone-sealant-coated denim pouch: (**a**) textile PIFA and (**b**) wideband monopole.

To test the waterproofing covers, both antennas in protective silicone-sealant-coated denim pouches were sprayed with water. The pouches proved to be waterproof, with water droplets forming on the outside (Figure 15). The presence of the water droplets resulted in further detuning of the PIFA (Figure 13), while they had no effect on the monopole (Figure 14).

Measurements have shown that the protective silicone-sealant-coating of the denim pouches has negligible effect on the shape of the radiation patterns in both textile antennas. For the PIFA the waterproofing cover reduced the antenna gain by 1 dB. The water droplets formed on the protective pouches after spraying with water had no influence on the antenna gain values. The waterproofing denim pouches offer excellent protection of both textile antennas from moisture, while the antennas remain flexible and wearable. However, the influence of additional dielectric layers has to be taken into account when designing the textile PIFA to avoid detuning. The loss of about 1 dB in the gain value for the PIFA is acceptable regarding the benefit obtained from waterproofing.

## 5. Resonant Textile PIFA as Moisture Sensor

The large detuning effect of moisture on the resonant textile PIFA led to the possibility of using it to sense moisture content [16]. As shown in Figure 5, increased moisture content shifts the antenna resonant frequency (and with it the bandwidth in which the input impedance is matched) to lower values. Furthermore, the measured gain (Figure 6) shows that the antenna still radiates at these new, lower resonant frequencies. This allows the implementation of two measurement setups in which a textile PIFA can be used as moisture sensor. It must be mentioned that in both measurement setups the PIFA has to be protected from other influences, such as mechanical deformations, which can cause detuning. Hence, the moisture sensor with the textile antenna is not for wearable, but for stationary, use.

### 5.1. Textile PIFA as Moisture Sensor in Transmission Mode

**Figure 16:**
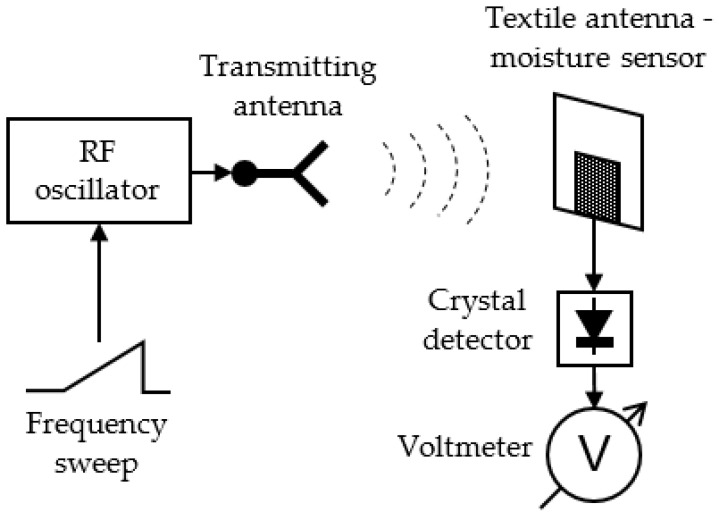
Textile antenna as moisture sensor, transmission mode setup [16].

The transmission mode exploits both the shift of the resonant frequency (and with it impedance matching bandwidth at the antenna input) and the shift of peak gain frequency towards lower frequencies with increased moisture content. The moisture measurement setup with textile antenna in transmission mode is shown in Figure 16.

**Figure 17:**
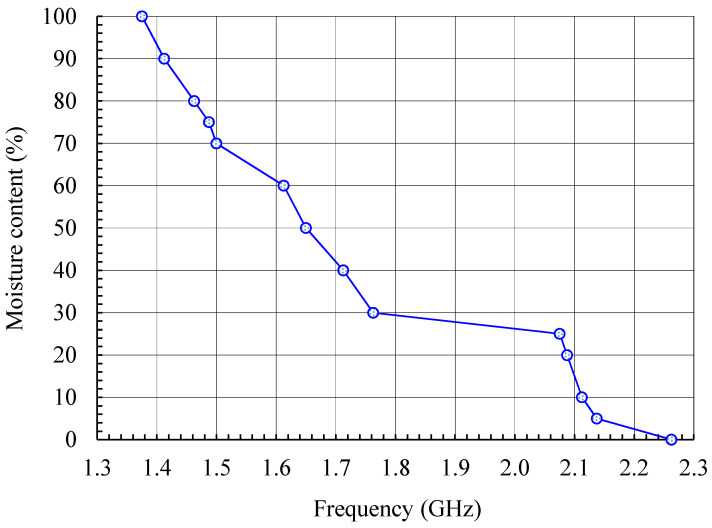
Calibration curve connecting the resonant frequency and moisture content for the textile PIFA. The markers indicate measured values.

A swept RF oscillator is used to feed a transmitting antenna. Its frequency is swept in the entire band in which it is expected that the textile antenna can be detuned due to moisture. As the detuning bandwidth of the textile antenna can be quite large, the transmitting antenna should be broadband, e.g., a conventional wideband horn or printed Vivaldi antenna. At the receiving end is the textile antenna which senses the moisture content. A crystal detector connected to the textile antenna output port is used to measure the voltage of the received signal. As the transmitted signal is swept in frequency, the detected voltage will change. According to the Friis transmission equation [8] (pp. 94–96), the largest absolute value of the received voltage will be obtained at the frequency at which the textile antenna has maximum gain and best impedance matching. From this frequency, we obtain the moisture content. The calibration curve connecting the resonant frequency and moisture content is shown in Figure 17. It is obtained from measured data in Figure 5.

### 5.2. Textile PIFA as Moisture Sensor in Reflection Mode

**Figure 18:**
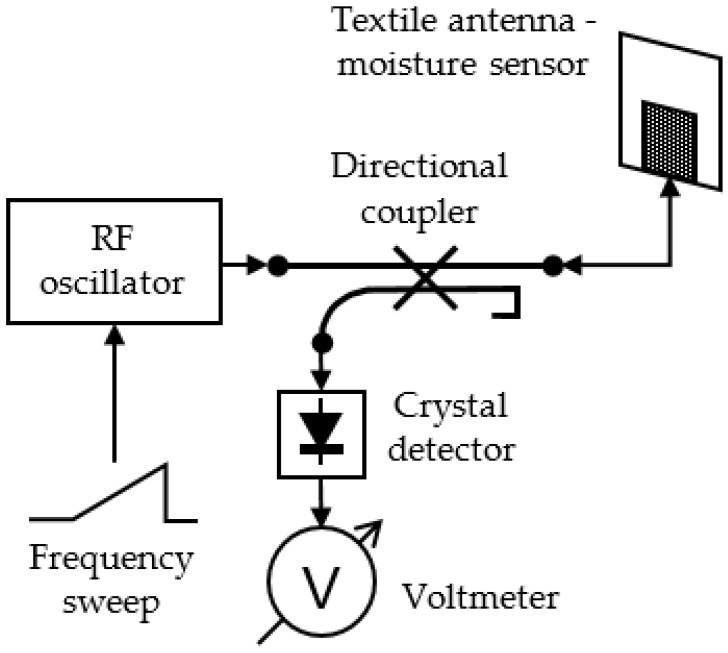
Textile antenna as moisture sensor, reflection mode setup [16].

The moisture measurement setup with textile antenna in reflection mode is more compact in relative to the transmission setup. The schematic is shown in Figure 18.

Only the textile antenna used for moisture sensing is placed in the space where moisture should be sensed and the reflection at its input port is measured. Here the textile antenna—moisture sensor—is excited from a swept RF oscillator. The wave reflected at the antenna input port is extracted by a directional coupler and sensed at the coupled port by a crystal detector. The antenna resonant frequency is determined from the minimum absolute value of the measured voltage, as at resonance the reflection is minimal. The calibration curve connecting the resonant frequency and moisture content is the same as for the transmission mode moisture measurement setup (Figure 17).

### 5.3. Discussion and Experimental Verification of Moisture Measurement

In transmission mode both the antenna impedance match and gain affect the received power level (and detected voltage), thus apparently transmission mode measurement should give more accurate results for measured moisture content. However, the received power given by the Friis transmission equation [8] (pp. 94–96) is proportional to the antenna gain and the factor (1 − |*S*_11_|^2^) which takes in account the antenna input impedance mismatch. The gain vs frequency plots (Figure 6) show broad and flat maxima for all moisture contents, while the factor (1 − |*S*_11_|^2^) transforms sharp notches at resonances in Figure 5 to flat maxima. As a consequence, the detection of the maximum received voltage and the frequency at which this maximum voltage occurs become more prone to error. This error then directly translates to an error in the measured moisture content, as was confirmed with measurements.

**Figure 19:**
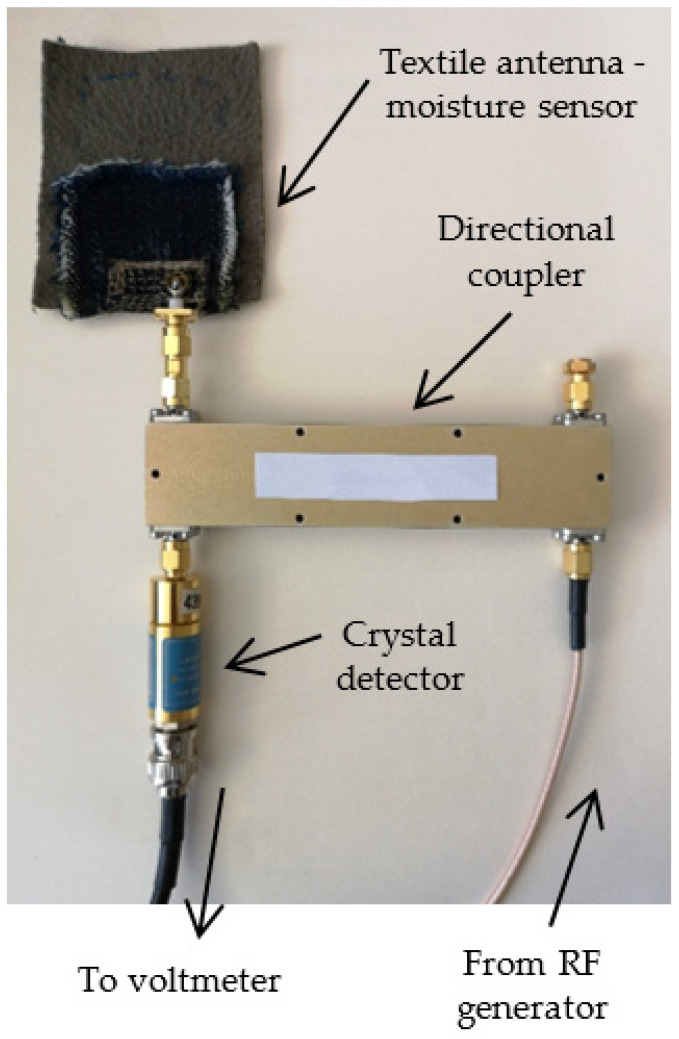
Experimental verification of reflection measurement mode setup.

On the other hand, the reflection mode directly uses the sharp reflection coefficient minima (Figure 5) for resonant frequency measurement which gives much more accurate results for measured moisture content. Therefore, the preferred method for moisture content measurement with the textile antenna would be the reflection mode setup. The whole setup can be assembled from low cost components [16], and it can be adapted to the frequency band which is most suitable for the user, i.e., to the available RF equipment. However, if some measurement automation is required, equipment with a possibility of automatic data recording and pairing of the frequency with corresponding detected voltage would be preferable. The photo of the reflection measurement mode setup is shown in Figure 19.

**Figure 20:**
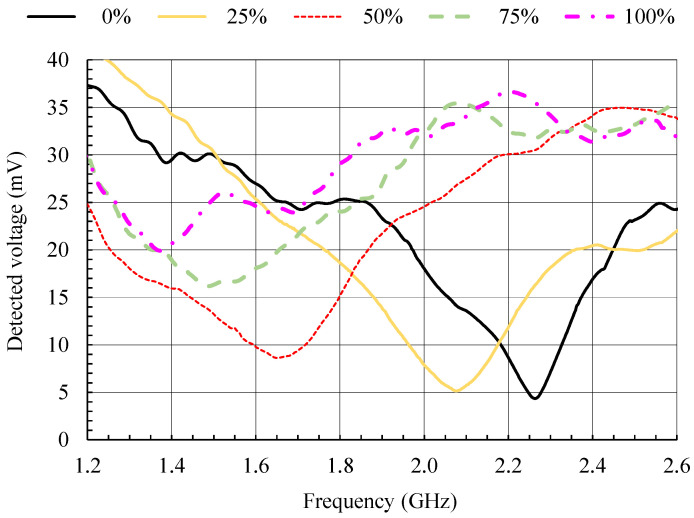
Average measured detected voltages in reflection measurement mode setup for ten measurement series and for moisture content of 0%, 25%, 50%, 75% and 100%.

To test the measurement setup in Figure 19, ten measurement series were performed, five for increasing moisture content from 0% to 100% and five for decreasing moisture content from 100% to 0%. The average detected voltages from all ten measurement series for moisture contents of 0%, 25%, 50%, 75% and 100% are shown in Figure 20. The frequencies at which the detected voltage minima occur are in good agreement with the resonant frequencies (*S*_11_ minima) in Figure 5.

**Figure 21:**
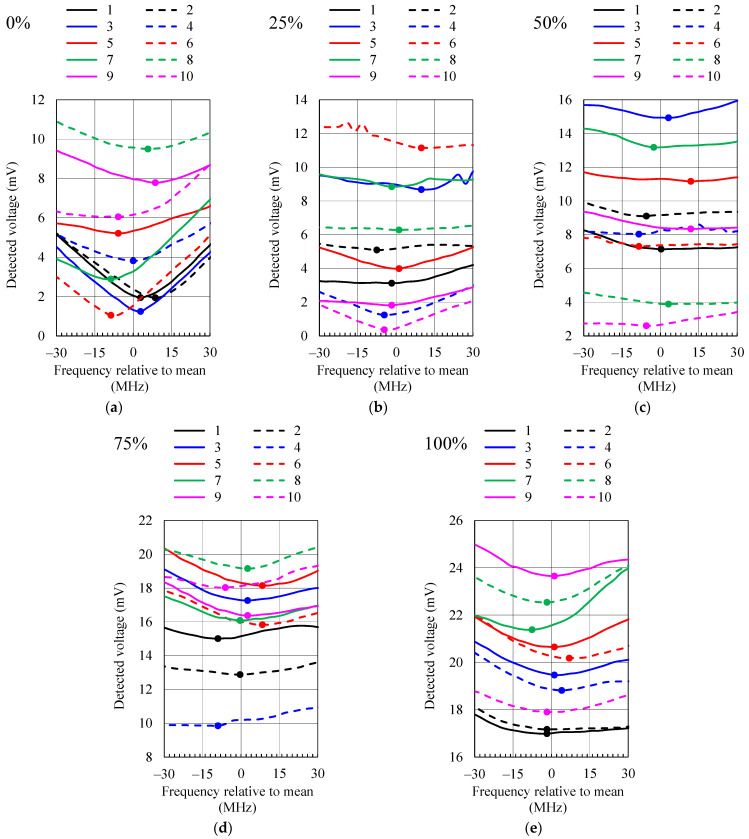
Measured detected voltage around the minima for ten measurement series and for moisture content: (**a**) 0%, (**b**) 25%, (**c**) 50%, (**d**) 75% and (**e**) 100%. The series for increasing moisture content are marked with odd numbers and plotted with solid lines, while the series for decreasing moisture content are marked with even numbers and plotted with dashed lines. The marker shows the minimum detected voltage.

As the moisture content is determined from the frequency at which the detected voltage minima occur, the repeatability of frequency measurement was tested. Each of the graphs in Figure 21 shows the detected voltage around the minimum for all ten measurement series at a fixed moisture content (0%, 25%, 50%, 75% and 100%). The series for increasing moisture content are marked with odd numbers and plotted with solid lines, while the series for decreasing moisture content are marked with even numbers and plotted with dashed lines. The mean frequency (zero frequency) on each of the graphs in Figure 21 corresponds to the frequencies at which the detected voltage minima in Figure 20 occur and to the frequencies given by the calibration curve in Figure 17.

Nevertheless, the magnitude of the detected voltage in Figure 21 varies between different series; the frequencies at which the voltage minima occur are grouped within 20 MHz or less. In Figure 21, the largest deviation of the measured frequency from the mean value is 11.9 MHz (moisture content 50%; series 5). Assuming a linear interpolation between the measured points on the calibration curve in Figure 17 and a worst-case scenario (steepest slope of the calibration curve: 0.4%/1 MHz), this largest deviation in frequency would result in an error of the measured moisture content of 4.8%. By taking the actual slope of the frequency–moisture curve at 50% moisture content, the error in the measured moisture content would be half of that value.

The estimated maximum error in moisture content measurement shows that the tested system does not provide highly accurate results, but this does not exclude it from applications where such errors are acceptable, e.g., moisture sensing in some warehouses, offices, or residential premises. However, taking in account that the PIFA was not designed to be a moisture sensor, but a part of on-body communication system, we see that the PIFA served well for demonstration of the moisture measurement concept. A textile antenna or resonator designed to be primarily moisture sensor would give better results. First of all, this antenna or resonator should be made less sensitive to mechanical deformations, such as bending and crumpling during handling and measurement (e.g., by using stiffer textile substrate), as mechanical deformations have also a detuning effect on textile antennas [4]. When determining the antenna dimensions, one should prevent the resonances of higher or lower operating modes and any multiple resonances in the frequency band of interest. Multiple resonances and overlapping operating modes should be avoided (e.g., by selecting appropriate antenna dimensions), not only for the dry antenna–resonator but also for the antenna at all moisture contents of interest. This will assure unique correspondence between the detuned resonant frequency and the moisture content.

## 6. Conclusions

The influence of moisture on two types of textile antennas, a resonant PIFA and a wideband monopole antenna, was experimentally examined. We considered possible applications in communications systems and the application of a resonant PIFA as moisture sensor. The main effect of moisture on resonant textile PIFA is detuning. Even a moisture content of less than 10% causes significant detuning and, consequently, impedance mismatch at the antenna input. A substantial decrease in PIFA gain value and some increase in cross-polarization levels occur at a moisture content greater than 50%.

The textile monopole antenna, due to its wideband characteristic, is less affected by moisture when compared to the PIFA. Good impedance matching is maintained in almost whole bandwidth and for all moisture contents up to 100%. For moisture content above 50% some increase in reflection at the monopole input is observed at the higher end of the bandwidth. An average gain reduction of 6 dB was observed for moisture content below 50%, while, above that value, the monopole gain shows notches of more than 10 dB at certain narrower bands.

It can be concluded that wideband textile antennas are less affected by moisture than the resonant antennas. A wireless communication system with a wideband textile antenna can maintain its functionality even at 100% moisture content in the antenna but with reduced communication range.

Nevertheless, as moisture can seriously impair the operation of both considered textile antennas in communication systems, covers of waterproofed textile were proposed as feasible and effective solutions to protect both considered antennas from moisture. The proposed waterproofing covers maintain the flexibility and wearability of both textile antennas without significant impact on the gain and radiation patterns.

The detuning effect of moisture on the textile PIFA and correspondence between the resonant frequency and moisture content make this antenna suitable as a moisture sensor. Two moisture measurement setups were tested. The first setup used the textile antenna in transmission, while the second considered only the reflection at the antenna input. Since the setup based on the antenna in transmission relies on maximum received signal which has flat maximum, while the reflection setup relies on sharp and narrow reflection minima at antenna resonances, the reflection method offered more reliable and accurate results for moisture content measurement, as well as satisfactory measurement repeatability.

## Data Availability

The data presented in this study are available on request from the corresponding author.
